# Research Note: Morphological and hormonal characterization of wild-derived Japanese quail (*Coturnix japonica*) strains

**DOI:** 10.1016/j.psj.2025.106036

**Published:** 2025-10-30

**Authors:** Yasuko Tobari, Sumire Hamasaki, Jun-ich Shiraishi, Prudence Nyirimana, Tatsuhiko Goto

**Affiliations:** a Laboratory of Animal Genetics and Breeding, Department of Animal Science and Biotechnology, School of Veterinary Medicine, Azabu University, Fuchinobe 1-17-71, Chuo-ku, Sagamihara City 252-5201, Japan; b Center for Human and Animal Symbiosis Science, Azabu University, Fuchinobe 1-17-71, Chuo-ku, Sagamihara, Kanagawa 252-5201, Japan; c Laboratory of Applied Biochemistry, Department of Animal Science, Nippon Veterinary and Life Science University, Kyonan-cho 1-7-1, Musashino City 180-8602, Tokyo, Japan; d Department of Life and Food Sciences, Obihiro University of Agriculture and Veterinary Medicine, Obihiro 080-8555, Hokkaido, Japan; e Research Center for Global Agromedicine, Obihiro University of Agriculture and Veterinary Medicine, Obihiro 080-8555, Hokkaido, Japan

**Keywords:** Backcross, Domestication, Hybrid, Strain difference

## Abstract

Two wild-derived strains of the Japanese quail were recently established by crossing wild males captured in Tokachi, Hokkaido, Japan, with females of a domestic strain. These new strains (W50 and W75) have approximately 50 % and 75 % wild genetic backgrounds, respectively. This study characterized morphological and hormonal traits of sexually mature male Japanese quail from a domestic strain and both wild-derived strains. Six morphological traits (tarsus length, exposed culmen length, bill width, bill depth, body mass, and cloacal gland volume) and plasma testosterone and thyroxine levels were analyzed. W75 males were significantly lighter than both W50 and domestic males and had shorter tarsi and smaller cloacal glands than W50 males. Hormone assays showed that W75 quail had lower circulating testosterone and thyroxine levels than W50 quail. Within-strain analyses revealed distinct correlation patterns: in domestic quail, tarsus length was positively associated with body mass, whereas in W75 quail, bill height correlated with bill width, and tarsus length with cloacal gland volume. These results indicate that genetic contributions from wild populations shape both morphological and hormonal profiles in Japanese quail. Wild-derived strains such as W50 and W75 provide valuable models for understanding the evolutionary and physiological consequences of avian domestication.

## Introduction

The Japanese quail (*Coturnix japonica*) is a small galliform bird native to East Asia with a distribution covering Japan, Korea, eastern China, Mongolia, and Sakhalin. The Japanese quail was first domesticated in Japan in the 17^th^ century for its vocalizations and then in the 20^th^ century for its egg production (Sano, 2009). Domestic quail are widely used for egg production and as laboratory animals due to their small size, rapid maturation, and ease of maintenance (Baer et al., 2015); however, wild quail populations in Japan are declining, and *C. japonica* is now classified as Near Threatened. Therefore, it is difficult to conduct research on wild quail in Japan.

Goto et al. (2023) established two wild-derived strains by crossing wild *C. japonica* males from Tokachi, Hokkaido, with females from a domestic strain. These strains (W50 and W75) have approximately 50 % and 75 % wild genetic backgrounds, respectively. Wild-derived strains provide unique opportunities to study genotype–phenotype relationships that are reduced or absent in domestic populations. In this study, we comprehensively characterized the morphology and hormone levels of male domestic and wild-derived (W50 and W75) strains. We hypothesized that W75 quail would exhibit phenotypes more similar to wild birds and distinct from domestic quail due to their higher proportion of wild ancestry. We also examined correlations among traits to better define strain-specific patterns. Our results revealed clear differences in both morphological and hormonal traits among the three strains, highlighting the influence of wild genetic contributions.

## Materials and methods

### Ethics statement

All experiments were approved by the Experimental Animal Committee of the Obihiro University of Agriculture and Veterinary Medicine (authorization no. 24-44) and followed the Animal Research: Reporting of *In Vivo* Experiments guidelines (https://arriveguidelines.org/arrive-guidelines).

### Subjects and housing

We obtained 24 male Japanese quail (domestic, n = 8; W50, n = 8; W75, n = 8) from a breeding colony at the Obihiro University of Agriculture and Veterinary Medicine. All birds were aged 220–626 days and sexually mature, as inferred by an enlarged cloacal gland, and their body mass ranged from 93.9 to 127.9 g. The quail were maintained under a long-day (16-h light:8-h dark) photoperiod, with the lights turned on at 05:00, and provided mixed feed for laying birds, which contained 17 % crude protein (Rankeeper; Marubeni Nisshin Feed, Tokyo, Japan), and water *ad libitum*. Each bird was marked with a silver wing identification tag. All birds were housed in individual wire cages (112 mm × 210 mm × 170 mm) in a single room, and had visual and auditory contact with other birds. We used eight birds from each strain (domestic, W50, and W75). The domestic strain was derived from a quail population used for egg production. The W50 strain was produced through natural mating of domestic female quail with wild males, and the W75 strain was produced by backcrossing W50 females with wild males (Goto et al., 2023).

### Morphological measurements

In March 2023, quail were caught and measured using digital calipers to determine their exposed culmen length (bill length), bill width, bill depth at 0.1-mm precision and tarsus length at 1-mm precision. We measured cloacal gland dimensions (length and width) and individual body weight (using a digital balance) at precision levels of 1 mm and 0.1 g, respectively. The cloacal gland volume was calculated from cloacal gland size measurements as 4/3 π ab^2^, where a = Long axis × 0.5 and b = Short axis × 0.5. All morphological analyses were based on data from 24 individuals (domestic, n = 8; W50, n = 8; W75, n = 8).

### Hormone plasma concentration measurements

Blood samples were collected from the branchial vein using a heparinized capillary tube (Thermo Fisher Scientific, Waltham, MA, USA) and then centrifuged at 3,000 *g* for 15 min at 4 °C to separate plasma from blood cells. The harvested plasma was kept at –80 °C until circulating hormone assays. Plasma total testosterone and total thyroxine (T₄) concentrations were determined for 23 males (domestic, n = 8; W50, n = 8; W75, n = 7) and 20 males (domestic, n = 7; W50, n = 7; W75, n = 6), respectively, via enzyme-linked immunosorbent assays (ELISAs).

For testosterone concentration measurements, we used enzyme immunoassay kits (Cat. No. ADI-900-065, Enzo Life Sciences, Farmingdale, NY, USA). Although the ELISA immunoassay kits and protocols used for testosterone measurements were validated previously and used in several avian studies, we first optimized them for Japanese quail plasma by testing two plasma dilutions (1:20, 1:40) and various concentrations (2 %, 2.5 %, 3 %, 3.5 %, 4 %, 4.5 %, and 5 %) of a steroid displacement reagent (SDR) to inhibit testosterone from binding to sex hormone-binding globulin or other proteins. Over several assays, we determined the optimal materials to be a 1:40 plasma dilution and 4 % SDR. Testosterone levels were measured in duplicate 100-μL quail plasma samples (1:40 dilution: 7 μL plasma + 7 μL 4 % SDR + 266 μL assay buffer). First, 100 μL of standard diluent was pipetted into the non-specific binding (NSB) and maximum binding (B0) wells of a 96-well plate, and 100 μL each of standard and diluted samples was pipetted into the respective wells. Then, 50 μL of antibody was added to each well except for the blank, total activity, and NSB wells. The plate was incubated for 1 h at room temperature on a plate shaker. Then, 50 μL of conjugate was added to each well except the blank and total activity wells, and the plate was incubated for 1 h at room temperature. Next, the plate was washed three times, 200 μL of *p*-nitrophenyl phosphate in buffer substrate solution was added, and the plate was incubated for 1 h at room temperature without shaking. After incubation, 50 μL of stop solution was added to halt the reaction, and the optical density at 405 nm (OD_405_) was read with 590-nm correction using an iMark microplate reader (Bio-Rad Laboratories, Hercules, CA, USA). The testosterone concentrations of experimental samples were calculated with reference to values from a standard curve drawn based on the OD of standard samples. The sensitivity and intra-assay variability of the assay were 7.81 pg/mL and < 3.28 %, respectively.

For total thyroxine concentration measurements, we used enzyme immunoassay kits (Cat. No. K050-H1, Arbor Assay, Ann Arbor, MI, USA). This assay has been previously validated and used in several avian studies. Thyroid hormones were extracted from 20 μL of room-temperature plasma in ethyl acetate. Liquid–liquid extraction was performed by adding 2 mL of ethyl acetate and vortexing for 5 min. The solvent layer was allowed to separate for 5 min at room temperature. Samples were frozen in a dry ice bath, and solvent solution collected from the top of the sample was transferred into a clean glass tube. The liquid–liquid extraction step was repeated twice for maximum extraction efficiency, combining the top layer of ether acetate solution in each iteration. Pooled solvent samples were dried at 40°C under nitrogen. The resulting pellet was stored at –30 °C to avoid compound degradation until the assay was performed.

On the day of the ELISA, the extract was redissolved at room temperature in 120 μL of the kit-specific assay buffer to a single-sample volume of 120 μL and final dilution of 1:6. Then, 100 μL each of the standard and sample were pipetted to designated wells, and 125 and 100 μL of assay buffer was pipetted to the NSB and B0 wells, respectively. Next, 25 μL of thyroxine conjugate was added to each well, and 25 μL of thyroxine antibody was added to all wells except the NSB wells. The plate was incubated at room temperature on a plate shaker for 1 h, after which the incubation solution was discarded and the plate wells were washed four times with 300 μL of wash buffer. Next, 100 μL of 3,3′,5,5′-tetramethylbenzidine solution was added to each well, and the plate was incubated at room temperature for another 30 min without shaking. The reaction was halted by adding 50 μL of stop solution to each well. The OD_450_ of the plate was read with 620-nm correction using an iMark microplate reader (Bio-Rad Laboratories). The thyroxine concentrations of experimental samples were calculated with reference to a standard curve drawn from the OD of standard samples. The sensitivity of the assay was 55.3 pg/mL.

### Statistical analyses

The data were analyzed using the Jamovi v2.3.28 software. We compared group means using one-way analysis of variance (ANOVA) followed by Tukey’s test, or the nonparametric Kruskal–Wallis test followed by the Dwass–Steel–Critchlow–Fligner test. Spearman correlation coefficients were used to analyze correlations between morphological and hormonal variables and to test how these phenotypes were integrated within each strain. Correlation analyses were conducted within each strain using individuals for which both morphological and hormonal data were available (domestic, n = 7–8; W50, n = 7–8; W75, n = 6–7). All statistical tests were two-tailed, and significance was evaluated based on a threshold of *P* < 0.05.

## Results

### Morphological and hormonal traits

The means ± standard deviation of the six measured morphological traits and two measured plasma hormone concentrations for each quail strain are shown in Table 1. Highly significant differences in body mass were detected among strains (ANOVA, F_2,21_ = 7.918, *P* = 0.003), with the W75 strain showing significantly lower body mass than all other strains (*P* < 0.01). Tarsus length also differed significantly among strains (ANOVA, F_2,21_ = 9.7692, *P* = 0.001), and was significantly shorter in W75 than in W50 (*P* < 0.001). Cloaca gland volume differed significantly among strains (ANOVA, F_2,21_ = 15.9067, *P* < 0.001) and was significantly lower in W75 than in W50 (*P* = 0.009) and the domestic strain (*P* < 0.001). There were no significant differences in beak height, width, or length among strains.

**Table 1:** Comparison of morphological traits and plasma hormone levels among wild-derived strains (W75 and W50) and domesticated strains.

Trait	Strain			*P*
	W75	W50	Domestic	
Body mass (g)	102.78 ± 10.43^a^	118.64 ± 5.23^b^	119.95 ± 11.87^b^	0.003
Beak height (mm)	5.33 ± 0.47	5.45 ± 0.91	5.36 ± 0.89	0.951
Beak width (mm)	5.69 ± 0.35	5.62 ± 0.656	6.20 ± 0.44	0.059
Beak length (mm)	13.11 ± 0.70	14.39 ± 1.43	14.47 ± 2.24	0.178
Tarsus length (mm)	25.75 ± 0.71^a^	27.38 ± 0.74^b^	26.50 ± 0.76^a,b^	0.001
Cloacal gland volume (mm^3^)	0.69 ± 0.47^a^	1.49 ± 0.43^b^	2.04 ± 0.53^b^	< 0.001
Testosterone (ng/mL)	5.73 ± 1.91^a^	26.39 ± 23.68^b^	12.55 ± 7.02^a,b^	0.006
Thyroxine (ng/mL)	1.25 ± 0.45^a^	1.80 ± 0.29^b^	1.64 ± 0.35^a,b^	0.039

Data are means ± standard deviation. Different letters within the same row indicate significant differences (*P* < 0.05).

There were significant differences in plasma total testosterone concentration among strains (Kruskal–Wallis test, χ^2^ = 14.4, *P* = 0.006, df = 2), with significantly lower concentrations in W75 than in W50 (Dwass–Steel–Critchlow–Fligner test, *P* < 0.003; Table 1). The plasma total thyroxine concentration differed significantly among strains (ANOVA, F_2,17_ = 3.96, *P* = 0.039) and was significantly lower in W75 than in W50 (*P* = 0.034; Table 1)

### Phenotypic correlations

**Fig. 1:**
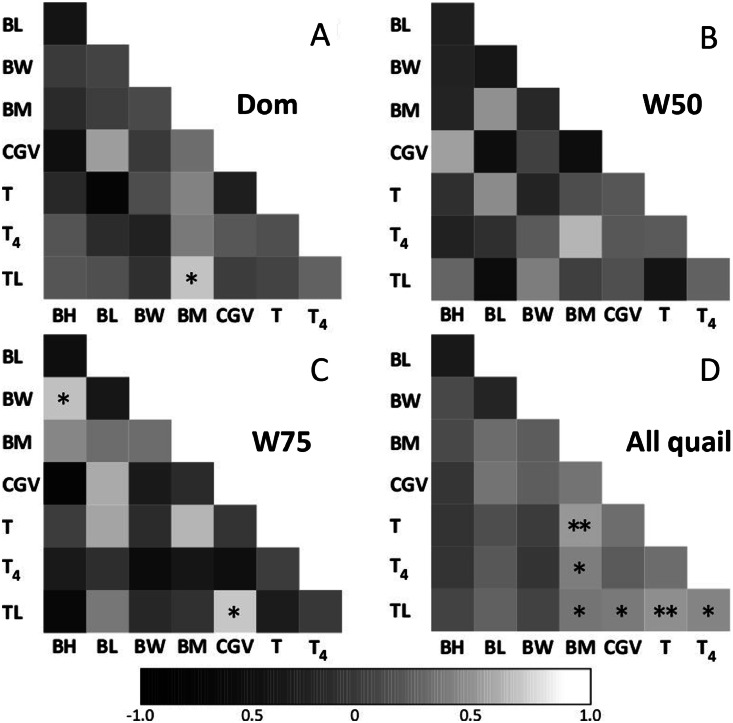
Spearman rank correlation analysis results for measured variables in (A) domestic quail, wild-derived quail with (B) 50 % and (C) 75 % wild genetic background, respectively and (D) all quail. BH, bill height; BL, bill length; BM, body mass; BW, bill width; CGV, cloacal gland volume; T, testosterone; T4, thyroxine; TL, tarsus length. Sample sizes for correlation analyses varied slightly depending on hormone availability: domestic (n = 7–8), W50 (n = 7–8), W75 (n = 6–7). Asterisks indicate significant correlations (*P < 0.05, **P < 0.01).

Tarsus length and body mass were positively correlated in the domestic strain (Fig. 1A). Bill height was positively correlated with bill width, tarsus length, and cloacal gland volume in W75 (Fig. 1C), but not in W50 (Fig. 1B). All quail showed significant positive correlations between body mass and plasma testosterone concentration, plasma thyroxine concentration, and tarsus length; and between tarsus length and cloacal gland volume, plasma testosterone concentration, and plasma thyroxine concentration (Fig. 1D).

## Discussion

We compared morphological and hormonal traits among male quail from domestic, W50, and W75 strains. Our results revealed distinct differences among the three strains. W75 quail were significantly lighter, with smaller cloacal glands than both domestic and W50 strains, indicating reduced body mass and secondary sexual development in the strain with the highest proportion of wild ancestry. In contrast, tarsus length and circulating testosterone and thyroxine levels were lower in W75 than in W50, but did not differ significantly between these strains and the domestic strain. These findings suggest that the effects of wild genetic contribution on phenotype are trait-specific, with some characteristics diverging from both domestic and W50 strains, while others remained comparable to those of domesticated birds.

In birds, domestication is typically associated with increased body mass, elongated body length, and elevated reproductive traits (Paulke and Haase, 1978; Chang et al., 2009, Tschirren et al., 2009; Prior et al., 2017; Endo et al., 2022). The lower body mass and cloacal gland size observed in W75 are consistent with the influence of wild ancestry, while the intermediate values of W50 suggest partial retention of domestic characteristics. Interestingly, the absence of differences in tarsus length and hormone levels between W75 and domestic quail indicates that changes related to domestication were not uniform across all traits, and that some morphological and endocrine features may be more resistant to genetic introgression from wild populations.

Trait correlation analysis revealed both strain-specific and broad associations, highlighting the influence of domestication and wild genetic contributions on the integration of morphological and endocrine systems. In domesticated quail, tarsus length and body mass were positively correlated, consistent with a report that artificial selection has favored coordinated growth in skeletal and somatic traits in broiler chickens to achieve uniform body size for meat production (Nosike et al., 2018). In contrast, W75 quail exhibited two distinct correlations: between bill height and width, and between tarsus length and cloacal gland volume. The former suggests developmental integration of craniofacial traits, while the latter indicates a functional link between structural body growth and reproductive status. To our knowledge, no previous studies have directly examined the relationships between skeletal traits and accessory sex organs in wild birds, although beak depth and width are positively correlated in many bird species due to a combination of mechanical constraints and ecological adaptation (Mallarino et al., 2011). Therefore, correlations between beak dimensions may reflect the retention of developmental or physiological constraints in wild quail populations. In contrast, no significant correlations were detected in W50, indicating that intermediate wild–domestic genetic backgrounds may disrupt trait associations. However, when all strains were analyzed together, consistent correlations emerged across quail strains, such that plasma testosterone and thyroxine concentrations were positively associated with body mass, while tarsus length was positively correlated with body mass, cloacal gland volume, and both plasma testosterone and thyroxine concentrations. These results indicate common physiological–morphological associations across Japanese quail, which may not be detectable when analyses are restricted to single lineages with limited sample size or reduced variance.

The strain-specific differences detected in this study emphasize the value of wild-derived strains as models for dissecting how domestication alters phenotypic integration, and more broadly, how evolutionary processes shape the covariation of morphology and physiology in birds. From a conservation perspective, the establishment of wild-derived strains such as W50 and W75 provides valuable genetic resources for understanding and preserving the diversity of Japanese quail. Wild quail populations in Japan are declining, and the species is now classified as Near Threatened. Comparative studies of wild-derived and domesticated strains could inform both conservation and breeding strategies. Future work should expand to include female quail and evaluate additional physiological traits such as stress responsiveness, metabolic rate, and seasonal reproductive plasticity. By integrating morphological, hormonal, and genomic approaches, such studies will contribute to a more comprehensive understanding of how domestication reshapes avian phenotypes and provide insights applicable to other domestic bird species.

## Declaration of generative AI and AI-assisted technologies in the writing process

During the preparation of this work the authors used Consensus, DeepL and OpenAI’s ChatGPT to assist in summarizing background literature and to improve the clarity and readability of the manuscript. After using this tool, the authors reviewed and edited the content as needed and take full responsibility for the final version of the manuscript.

## CRediT authorship contribution statement

**Yasuko Tobari:** Conceptualization, Data curation, Formal analysis, Funding acquisition, Investigation, Methodology, Project administration, Supervision, Validation, Visualization, Writing – original draft. **Sumire Hamasaki:** Formal analysis, Investigation, Writing – review & editing. **Jun-ich Shiraishi:** Investigation, Writing – review & editing. **Prudence Nyirimana:** Investigation, Writing – review & editing. **Tatsuhiko Goto:** Conceptualization, Data curation, Formal analysis, Investigation, Visualization, Writing – review & editing.

## Disclosures

The authors declare no conflict of interest.

## Data Availability

The data that support the findings of this study are available from the corresponding author upon reasonable request.
